# A Rapid Spin Column-Based Method to Enrich Pathogen Transcripts from Eukaryotic Host Cells Prior to Sequencing

**DOI:** 10.1371/journal.pone.0168788

**Published:** 2016-12-21

**Authors:** Zachary W. Bent, Kunal Poorey, Annette E. LaBauve, Rachelle Hamblin, Kelly P. Williams, Robert J. Meagher

**Affiliations:** 1 Systems Biology Department, Sandia National Laboratories, Livermore, California, United States of America; 2 Biotechnology and Bioengineering Department, Sandia National Laboratories, Livermore, California, United States of America; Cornell University, UNITED STATES

## Abstract

When analyzing pathogen transcriptomes during the infection of host cells, the signal-to-background (pathogen-to-host) ratio of nucleic acids (NA) in infected samples is very small. Despite the advancements in next-generation sequencing, the minute amount of pathogen NA makes standard RNA-seq library preps inadequate for effective gene-level analysis of the pathogen in cases with low bacterial loads. In order to provide a more complete picture of the pathogen transcriptome during an infection, we developed a novel pathogen enrichment technique, which can enrich for transcripts from any cultivable bacteria or virus, using common, readily available laboratory equipment and reagents. To evenly enrich for pathogen transcripts, we generate biotinylated pathogen-targeted capture probes in an enzymatic process using the entire genome of the pathogen as a template. The capture probes are hybridized to a strand-specific cDNA library generated from an RNA sample. The biotinylated probes are captured on a monomeric avidin resin in a miniature spin column, and enriched pathogen-specific cDNA is eluted following a series of washes. To test this method, we performed an *in vitro* time-course infection using *Klebsiella pneumonia*e to infect murine macrophage cells. *K*. *pneumonia*e transcript enrichment efficiency was evaluated using RNA-seq. Bacterial transcripts were enriched up to ~400-fold, and allowed the recovery of transcripts from ~2000–3600 genes not observed in untreated control samples. These additional transcripts revealed interesting aspects of *K*. *pneumoniae* biology including the expression of putative virulence factors and the expression of several genes responsible for antibiotic resistance even in the absence of drugs.

## Introduction

Next-generation sequencing of RNA (RNA-Seq) has emerged as a powerful new technology with wide application space in diverse fields such as cancer research and plant science [1, 2]. One area in which RNA-Seq has great, but currently unmet, potential is in the study of host-pathogen interactions. Understanding the correlated gene expression of both the host and pathogen together in different tissues and time points during an infection would significantly improve our understanding of the dynamic host-pathogen relationship. Current RNA-Seq methodology is well-suited to measuring the genes expressed by the host in response to infection with a pathogen [3–5]. However, the genes expressed by the pathogen while invading host tissue and evading the host immune response are much harder to discern. This is because it is extremely difficult to obtain sufficient pathogen transcripts from an infected host sample, particularly early in the infection when the pathogen is least abundant, but during which time the pathogen may be actively adapting to the host environment, or evading host immune response. Typically host transcripts outnumber pathogen transcripts by well over 100 fold [6–8], meaning that using a standard RNA-seq library prep to sequence the pathogen transcripts in a mixed sample can be an expensive and computationally wasteful proposition [9, 10].

The large differential between host and pathogen transcript numbers was a known problem facing attempts to profile pathogen expression in microarray experiments [11, 12]. This problem also affects RNA-seq, even though the number of reads and depth of coverage is very high. Enrichment of low-abundance pathogen transcripts is beneficial to achieve sufficient depth of coverage to discern the dynamics of the pathogen transcriptome. We do note that in certain models (*e*.*g*. experiments performed at high MOI [13], or with a physical separation of pathogen-infected cells [14]) enrichment may not be necessary, and in that event enrichment is undesirable to avoid introduction of any unnecessary bias.

If enrichment is needed, pathogen transcripts can be separated from host transcripts at three stages prior to sequencing, each of which has demonstrated some level of success. Recent work has demonstrated the utility of an up-front separation of bacteria from the host tissue; however, this method still benefits from additional separation at the RNA stage for the depletion of host transcripts [15]. Another recent study demonstrates fluorescence activated cell sorting (FACS) of infected cells prior to sequencing to enrich for an internalized, GFP-expressing bacterial pathogen [14]. Generalizing this approach requires a discernible fluorescent marker of infection, access to a FACS instrument, as well as developing sample prep and sorting techniques for each new infection model. Several methods (some commercially available as kits) are depletive, removing RNA or cDNA classes based on their abundance (host and ribosomal RNAs) or sequence (ribosomal RNAs). These approaches, *e*.*g*. Ribo-Zero epidemiology [16], microbeEnrich [17], depletion of abundant sequences by hybridization (DASH) [18] and hydroxyapatite chromatography [19], act negatively and do not specifically select for pathogen RNAs. These approaches are overall beneficial by reducing the most abundant transcripts (most notably host ribosomal RNA) but may still be insufficient on their own when the bacterial load is very low. Despite the inclusion of RNAse inhibitors with these kits, any extended processing or handling of RNA at the bench top can lead to degradation of RNA, biasing the sample against unstable transcripts [20]. These kits typically require a large quantity of starting material (> 1 μg) and may also enrich for bacterial and viral transcripts other than the specific pathogen that is being studied. The final stage prior to sequencing in which bacterial transcripts can be enriched is after the RNA has been converted to cDNA. Working with samples at this stage has several advantages including the increased stability of cDNA and the need for less starting material.

We have previously reported an enrichment strategy for pathogen transcripts based on hybridization with biotinylated capture probes that are generated enzymatically from genomic DNA of the pathogen [7]. Processing was performed in a custom-built microfluidic system that handled one sample at a time, and furthermore required significant engineering expertise to construct and operate, which put the technique outside the capabilities of many microbiology laboratories. We report here a method that significantly improves upon our previous enrichment strategy by increasing the throughput to 12 samples simultaneously and decreasing the time required. Furthermore, the new protocol utilizes only commercial, off-the-shelf equipment that is commonly available in molecular biology laboratories. This hybridization-based method specifically enriches for the pathogen of interest at the cDNA level and enables sequencing of both the host and pathogen transcriptome from the same sample with minimal sample input requirements. We demonstrate our approach here with an *in vitro* infection study performed with a multi-drug resistant strain of *Klebsiella pneumoniae*, which is emerging as a significant cause of nosocomial infections.

## Methods and Materials

### Bacterial strains, cells, and growth conditions

*Klebsiella pneumoniae* strain ATCC BAA-2146 [21, 22] was obtained from ATCC and routinely grown on LB agar or in LB broth (Gibco) at 37°C with shaking. P388D1 murine macrophage cells were also obtained from ATCC (ATCC® CCL-46™) and cultured in RPMI supplemented with 10% fetal bovine serum. Cell culture and infections were performed at 37°C in an atmosphere of 5% CO_2_.

### Infection of murine macrophage cells

**Fig 1:**
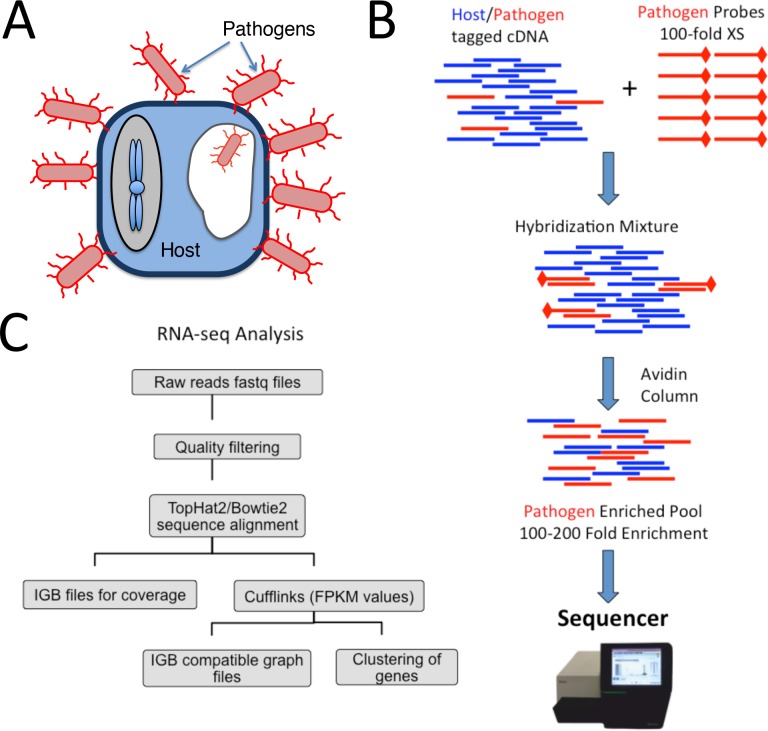
Schematic workflow of capture protocol and analysis. (A) Infection of pathogen (*K*. *pneumoniae*, red) to host P388D1 murine macrophage cells (blue). Non-cell associated bacteria are washed away prior to RNA extraction, such that the bacteria are primarily associated with host cells (both externally and internalized). (B) Capture protocol workflow where host pathogen cDNA with Illumina-compatible adapters is mixed with pathogen-specific capture probes for overnight hybridization. The hybridized mixture is then passed through a biotinylated avidin column to enrich for pathogen transcripts, prior to Illumina library construction. (C) RNA-seq workflow of YAnTra which was used to analyze RNA seq raw data where: Raw reads were quality filtered, aligned to reference genome, and processed with cufflinks to obtain gene expression.

P388D1 murine macrophages were grown in 6-well plates for two days to form a confluent monolayer. Cultures of *K*. *pneumoniae* were grown overnight and then sub-cultured into fresh medium and grown to mid-log phase. Concentrations of bacteria and macrophages were determined and the P388D1 cells were infected within the 6-well plates in triplicate at MOI (multiplicity of infection) of 0.1, 1, 10, or 100. The plates were centrifuged at 500 × g for 5 minutes to enhance adherence and then placed back in the incubator for 1 hr in the first experiment. In the second experiment the same procedure was followed using an MOI of 10 and incubation periods of 2, 4, 8, or 24 hr. After incubation, the RPMI medium was removed and the cells were washed twice with 37°C PBS to remove non-adherent bacteria, resulting in primarily macrophages with externally adhered and internalized [23] bacteria (Fig 1A). Cells were lysed and RNA was preserved by adding 1ml of RNAzol (Molecular Research Center, Inc.) to each well. The contents of each well was then transferred to 2 mL cryo-tubes and frozen at -80°C until the RNA extraction was performed.

### RNA extraction and cDNA Synthesis

Samples were thawed on ice. 400 μL of molecular biology grade water was added to each sample, and samples were mixed. After a 15-minute incubation at room temperature, samples were centrifuged at 4°C for 15 minutes at 16,000 × g. The bulk (800 μL) of the aqueous phase was transferred to a new tube and mixed with an equal volume of 100% ethanol. RNA was extracted with the Direct-zol kit (Zymo Research) according to the manufacturer’s instructions. RNA concentrations were determined by Qubit (Life Technologies), purity (A_260_/A_230_ and A_260_/A_280_) was determined by Nano Drop (Thermo), and the RNA integrity number for the total RNA was obtained by BioAnalyzer (Agilent). The RNA was next fragmented using the NEBNext Magnesium RNA Fragmentation Module [24] with a 3 minute incubation at 94°C followed by a cleanup step using the RNA Clean & Concentrator-5 (Zymo Research). Double-stranded, tagged cDNA was generated from 50 ng of fragmented RNA by the Peregrine method as previously described [7, 25].

### Enrichment of *K*. *pneumoniae* transcripts

*K*. *pneumoniae* was grown overnight in LB broth at 37°C with shaking. 1.5 mL of cultured bacteria was then collected by centrifugation and total genomic and plasmid DNA extracted using the DNeasy Blood and Tissue kit (Qiagen). Biotinylated *K*. *pneumoniae* probes were generated from the genomic and plasmid DNA using the BioPrime DNA Labeling System (Life Technologies) according to manufacturer’s instructions. The hybridization reaction was set up with 20 ng of double-stranded tagged cDNA mixed with 2 μg of probes and then dehydrated using a vacuum centrifuge and re-suspended in 10 μL of hybridization buffer (NimbleGen). The hybridization mixture was then denatured on a thermocycler at 95°C for 5 min followed by a 16–18 hr (overnight) incubation at 60°C. Monomeric avidin agarose (60 μL) (Pierce) was packed into Micro-Spin columns (Pierce) by 30 s of centrifugation at 600 × g. All subsequent steps were carried out in a 60°C incubator, with all buffers and equipment pre-warmed in the incubator for at least 2 hr. The packed column was washed twice with 200 μL of 0.1 M PBS and then the hybridized sample was applied directly to the packed resin. After 5 min incubation the sample was washed 15 times with 200 μL 2X Stringent Wash buffer (NimbleGen). The probe-bound sequences were then eluted off the column twice with 25 μL of elution buffer (Pierce). The eluted fraction was cleaned and concentrated to a final volume of 13 μL using 1.8X volumes of AMPure XP beads (Beckman Coulter). The enrichment protocol is illustrated schematically in Fig 1B.

### Final Library Preparation and Sequencing

A qPCR assay was performed on the captured cDNA samples to determine the correct number of cycles required to generate the final sequencing library as well as to assess the efficacy of normalization and enrichment [25]. Typically, higher C(t) values indicate greater depletion of host transcripts. The final library was created by PCR amplifying the samples with the full length sequencing adapters and custom 9-mer barcodes [25]. Following PCR, the samples were cleaned and size selected in a two-step cleanup using 0.75 volumes of AMPure XP beads followed by 0.15 volumes to achieve a final library with an average size of ~330 bp. Libraries were combined with 12 samples each, in equal amounts and concentrated using the DNA Clean & Concentrator-5 (Zymo Research). Prior to sequencing the final libraries were quantified using qPCR. Non-captured samples were prepared by diluting the double-stranded cDNA 100 fold and then adding sequencing adaptors and barcodes with 12 cycles of PCR. Combined libraries were sequenced on an Illumina MiSeq using 150-cycle kits and custom 9 base index read. Samples were loaded at 18 pM and 151 base single-end reads were obtained. All raw sequencing data has been deposited in the NCBI Sequence Read Archive (SRA) with the accession number PRJNA317373.

### RNA-Seq Analysis

The sequencing data was analyzed by our in-house YAnTra software pipeline (Yet Another Transcriptomics pipeline). The fastq files obtained from the MiSeq were analyzed by a previously described Perl script to filter out low-complexity or low-quality sequences, and parts of primer sequences [19, 26]. Briefly: sequence quality filtering is performed by a custom Perl script which removes internal barcodes and trims low quality fragments from the reads, fragments of primers used for the library construction and regions of low quality through Dustmasker. After applying these filters, reads shorter than 30 bp or with an overall quality score of less than 30 are filtered out. The quality filtered reads are then aligned to the mouse or *K*. *pneumoniae* reference genome using TopHat2 [27] and Bowtie2 [28] producing sam/bam files for alignment maps. The reference genome sequence and gene annotations for *K*. *pneumoniae* strain BAA-2146 were obtained from our previous study [22] [Genbank accession numbers CP006659.1, CP006660.1, CP006661.1, CP006662.1, and CP006663.1 corresponding to the chromosome and four plasmids]. Cufflinks [29] was used to analyze sam/bam files to get normalized expression values for genes as FPKM (Fragment Per Kilobase of exon per Million fragments mapped) values. A schematic of the YAnTra pipeline is shown in Fig 1C.

## Results and Discussion

The recently sequenced *Klebsiella pneumoniae* strain ATCC BAA-2146 (Kpn2146) was the first isolate in the United States found to encode the NDM-1 metallo-β-lactamase, making it resistant to carbapenem antibiotics, a class of broad-spectrum antimicrobials “of last resort” that are typically effective against other multi-drug resistant bacteria [30, 31]. Carbapenem-resistant *Enterobacteriaceae* (CRE) such as Kpn2146 represent a potential public health crisis [32, 33], yet *K*. *pneumoniae* has historically been labeled an opportunistic pathogen. Compared to more highly pathogenic bacteria, relatively little work has been done to elucidate the mechanisms it uses in host interaction. RNA-Seq of bacteria during an infection represents a novel way to rapidly increase knowledge about the genes bacteria express during infection and could be useful in determining key virulence factors [34].

To our knowledge, the complete transcriptional profile of opportunistic pathogens such as *K*. *pneumoniae* has not been studied using RNA-seq within the context of infection models, as opposed to pure culture [35]. We thus chose to demonstrate the efficacy of our bacterial transcript enrichment method in a preliminary study in which we infected a murine macrophage cell line with Kpn2146 at infectious doses ranging over four orders of magnitude as well as observing a 24-hour time course infection at a more standard infectious dose (MOI = 10). Because free-swimming bacteria are washed away, the signal we examine comes from bacteria that are primarily associated with the host cells or possibly attached to the plate. (Note that gentamicin treatment, a standard protocol to remove extracellular bacteria in tissue culture infections, is not feasible because this strain of *K*. *pneumoniae* has high-level resistance to gentamicin.) Our enrichment method is based on the hybridization of biotinylated probes against the bacteria of interest, selecting bacterial cDNA from the mixed host/pathogen cDNAs [6]. Probes were generated without bias from both strands of bacterial genomic DNA so that every bacterial transcript has a corresponding probe. As illustrated in Fig 1B, probes are hybridized with the mixed host and pathogen cDNA at 100-fold excess ensuring that the complete dynamic range of bacterial transcripts can by captured. In a scenario where host transcripts outnumber pathogen transcripts 100-to-1, the capture probes are present in approximately 10^4^-fold excess relative to bacterial transcripts, ensuring high capture efficiency. The hybridization mixture is passed through monomeric avidin-packed spin columns to bind all biotinylated probes, including those probes with bound cDNA transcripts. Through a series of washes the non-bound host transcripts are depleted and then the pathogen-enriched pool can be eluted off the column using a concentrated biotin buffer. The final sequencing library is created by PCR amplifying this pool to add full-length Illumina sequencing adaptors and indexes.

### Characterization of capture enrichment

**Fig 2:**
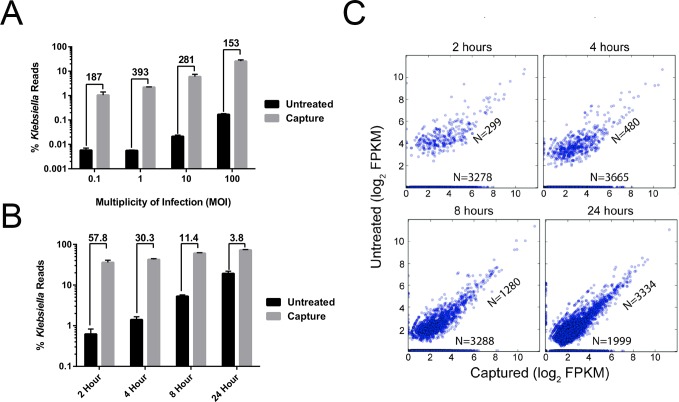
Characterizing performance of capture enrichment. Comparison of transcriptomics coverage over the captured and uncaptured samples for the infection time course. (A) Percentage of reads mapping to *K*. *pneumoniae* in the captured *vs* untreated controls in 1-hour infections at different MOI. (B) Percentage of reads mapping to *K*. *pneumoniae* in the captured *vs* untreated controls at different time points of an infection with MOI of 10. In both (A) and (B) the numbers above each group of bars refers to the fold-enrichment achieved in the captured *vs* untreated controls. Error bars refer to the standard deviation of percentage of reads from the triplicate infections. (C) Scatter plots of log_2_(FPKMs) for all genes comparing captured and uncaptured samples. The figures are marked by two numbers which show number of genes measured in both capture and uncaptured and genes, which were only measured in captured samples (along the x-axis). *K*. *pneumoniae* BAA-2146 has 5653 protein-coding genes and 190 RNA genes. The triplicate runs have been combined in each panel.

In the first experiment a short 1-hour infection was performed at 4 different multiplicities of infection (MOI) ranging from very low (0.1) to very high (100). In the second experiment, a time course was undertaken examining enrichment of Kpn2146 transcripts at two, four, eight and 24 hours post-infection. In each case we show consistently high levels of enrichment, especially evident in both the shorter time and lower MOI infections where the bacteria is less abundant. As shown in Fig 2A, in the one-hour infection, Kpn2146 transcripts were enriched by approximately 150- to 400-fold compared to the non-captured control. We also observed a significant enrichment of reads aligning to the bacterial genome by applying the capture protocol to RNA-seq libraries at all time points from the infection (2, 4, 8, and 24 hours), as shown in Fig 2B. No hybridization-based protocol is 100% selective for its target, because off-target hybridization can occur. In the case of an infection model the host RNA is such an overwhelmingly large fraction of the total RNA that even a highly selective capture protocol with stringent hybridization conditions and washes will result in carryover of host-derived RNA. The mapping of reads to the mouse genome was uniform in the uncaptured samples reflecting no bias. After capture the percentage of host reads decreased with increasing time of infection ranging between 38.7–19.9%.

To verify that the capture is unbiased, we first determined that Kpn2146 reads had a similar distribution over the transcriptome with or without the capture protocol. We compared the normalized expression values measured using cufflinks across the samples. A plot of FPKM values for each sample (S1 Fig) shows that the captured samples have a more complete representation of the pathogen transcriptome than non-captured samples, both in terms of the numbers of reads and the range of gene expression. This is also evident in Fig 2C, which compares normalized gene expression for all observed genes at each time point of infection in the captured *versus* uncaptured libraries. Points lying along the horizontal axis are genes that are observed in the captured library, but not observed in the uncaptured library. The number of genes observed in the captured library is more than 10-fold higher compared to the uncaptured library for the earliest infection time point (2 hours), which indicates that the capture technique is revealing genes that could not be observed by a standard RNA-seq protocol. For genes that are observed in both the captured and uncaptured libraries, the expression pattern of the genes are well correlated (R^2^ ~ 0.83–0.98) for captured *versus* uncaptured libraries, as indicated by the clustering of genes along the *y* = *x* lines in Fig 2B. This demonstrates that the capture protocol increases the sensitivity of RNA-seq without significantly distorting the gene expression levels, consistent with the observation made in our previous study with the lower-throughput version of our capture technique [7]. Uncaptured libraries are biased toward highly expressed genes at early time points in the infection; this bias decreases as the infection progresses. One simple explanation for this phenomenon is that growth of the bacteria over the time course of the infection leading to more bacterial RNA in the sample (up to 68% of total reads by the final time point). With the capture protocol the total reads mapping to bacterial transcripts increases by over two orders of magnitude, which makes it possible to measure a wider range of gene expression in the samples. Both captured and uncaptured samples show an increase in the number of activated genes during the course of the infection. The efficiency and sensitivity of RNA-seq for pathogens is significantly increased by the capture method.

To further assess reproducibility of the technique, and to address the possibilities of bias or probe saturation for highly expressed transcripts, we performed capture enrichment in triplicate using RNA isolated from three pure cultures of Kpn2146. As expected we found similar representation of genes in these libraries with and without the capture approach. A composite scatter plot showing FPKM for RNA-seq with and without the capture protocol with all three replicates combined is presented in S2 Fig. As with the infection models, the points clustered along the *y = x* line, with no deviation from this line for highly expressed genes (probe saturation would appear as a deviation from this line, with reduced representation of highly expressed genes following capture).

Additional visualizations of the sample-to-sample reproducibility and bias associated with capture are presented in S3–S5 Figs. These figures allow direct comparison of individual replicates from the pure culture experiment and a subset of the infection experiments, in the form of an array of scatter plots of FPKM allowing comparison of each individual replicate against each other replicate (S3 Fig), and “all *versus* all” heat maps showing distance (the inverse of similarity or correlation) between replicates of selected data sets (S4 and S5 Figs). To summarize these supplemental figures: the captured and uncaptured data sets from the pure culture experiments are highly similar to one another. The individual replicates of each particular condition tend to cluster together; *i*.*e*. are most similar to each other. The infection experiments form a separate cluster that is highly dissimilar to the pure culture experiments. Within each condition, the captured and uncaptured data sets appear dissimilar to each other due to the significantly different representation of the bacterial transcriptome in the captured data sets.

### Survey of gene expression in *K*. *pneumoniae* infection of murine macrophage cells

**Fig 3:**
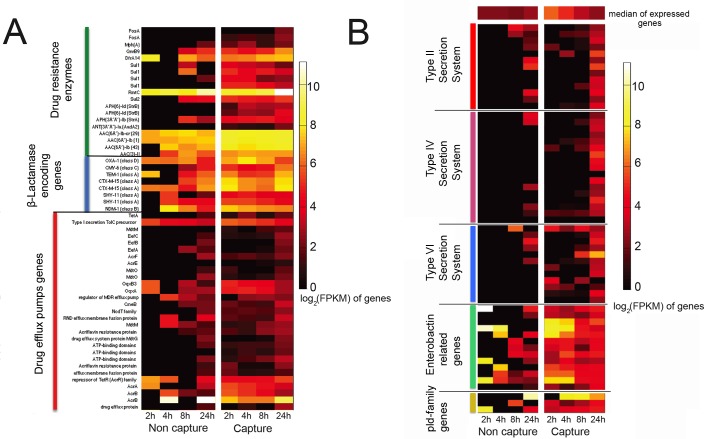
Dynamics of gene expression during infection. Heatmap representation of gene expression (log_2_(FPKMs)) for A) genes involved in antibiotic resistance over the time course of infection. This includes drug resistance enzymes, β-Lactamase encoding genes and drug efflux pump encoding genes. Genes are clustered according to the annotation class shown on the left. B) gene expression (log_2_(FPKMs)) pattern for genes putatively associated with virulence in *K*. *pneumoniae*. Genes are clustered according the annotation class shown on the left. For panel B of the heat map, a list of gene names in the order in which they appear is provided in S1 Table.

Kpn2146 is notable for its large arsenal of antimicrobial resistance genes as well as mutations in drug target sites and efflux pump regulatory elements [22], giving rise to a “pandrug-resistant” phenotype (*i*.*e*. resistant to all 34 drugs and drug/inhibitor combinations tested in a standard automated susceptibility testing panel for Gram-negative bacteria; the results are available at https://www.atcc.org/~/media/BA6C8F7C7C4C4649B2AEF501E51D76B8.ashx, collected 9/6/2016). Expression of genes associated with antimicrobial resistance over the time course of the infection are presented in the heat map in Fig 3A. Genes encoding drug resistance enzymes have been grouped together, with β-lactamase encoding enzymes as a subgroup, and multidrug efflux pumps grouped separately. Due to the low number of reads mapping to the bacteria in the uncaptured libraries, the gene expression data is sparse, whereas in capture samples the expression profile is visible for the whole time course. Expression of β-lactamase-encoding genes, including *bla*_NDM-1_, was high at all time points for all the samples, even though antibiotics were absent, consistent with previous studies [36]. Follow-up studies are planned to determine which, if any, of the resistance determinants (including efflux pumps) show inducible expression in the presence of antimicrobial drugs. We note further that 19 of the drug resistance genes are encoded on the three large plasmids of Kpn2146, along with numerous other genes associated with stress and survival. Our approach, generating probes directly from the total DNA of the isolate, allows us to enrich for transcripts regardless of plasmid or chromosomal origin. S6 Fig illustrates mapping of reads to the plasmid pNDM-US at the first and last time points, illustrating that the plasmid shows the same trend as the chromosome, of enriching for low-expressed genes.

Several putative virulence factors have previously been described in *Klebsiella pneumoniae* [37], of which capsule synthesis (*cps* genes) is the best characterized [38]. Kpn2146 does not display a hypermucoviscous phenotype, and lacks the *rmpA* regulator of capsule expression frequently observed in virulent *Klebsiella pneumoniae* infections [39, 40]. Heat maps showing gene expression for several other putative virulence factors are shown in Fig 3B [37, 41, 42]. Of these systems, genes associated with the siderophore enterobactin are highly expressed at early time points. Iron acquisition systems are well-known factors for *in vivo* growth of pathogens [43, 44], and enterobactin is the only siderophore we identified in the Kpn2146 genome. Improved coverage across the entire cluster of enterobactin-related genes is observed in the captured data set, compared to incomplete coverage in the non-captured data set. The Type II, Type IV (conjugation) and Type VI secretion systems all have relatively low expression early in the infection, although particularly for the Type VI system, the capture data set reveals low-level expression among several components of the systems that are not visible in the non-captured data set. Among the phospholipase genes, we observe the highest level of expression of *pld2* (the top row of the pld-family genes in the heat map), although *pld1* and *pldA* are expressed as well. The bacterial phospholipases are generally believed to play a role in membrane disruption and cell invasion by Gram-negative bacteria [45, 46]. The *pld1* gene was previously identified as a virulence factor in *K*. *pneumoniae* [37] although we are not aware of specific roles elucidated for *pldA* and *pld2* in virulence of *K*. *pneumoniae*.

Kpn2146 has 11 identified genomic islands [22]. Genes encoded within genomic islands are frequently associated with adaptation and pathogenicity [47, 48]. The gene expression profile within genomic islands over the time course of the infection and for both captured and uncaptured libraries is shown in S7 Fig. The capture method reveals that certain genomic islands (Kpn23SapB, Kpn40GuaA, Kpn16Fis) have higher gene expression than others at the 24 hour time point of the infection (S7 and S8 Figs).

For the sake of researchers who wish to analyze our data from the infection experiments for additional features, we provide as supplementary information tables of the read counts (S2 Table) and FPKM (S3 Table), as well as the corresponding annotation file (S4 Table). These tables provide data from each replicate of the time course and MOI experiments described above. The raw sequencing reads are provided in the NCBI Sequence Read Archive (accession number PRJNA317373).

We note that the host transcriptome can be profiled in an unbiased fashion from the original uncaptured library, allowing parallel analysis of the host and pathogen. We do not belabor the host results here, as this preliminary study was performed in a cell line which is not an accurate model of host behavior, *versus* an animal model or even primary cell model. The capture methodology is expected to enable such parallel host- and pathogen RNA-seq (or “Dual RNA-seq”) experiments in more complex models, with unprecedented resolution of the pathogen transcriptome. While this study was performed with a bacterial pathogen, based on our previous work [7] we believe viral transcriptomes could be captured using this technique as well.

### Pathogen transcript enrichment protocol

The entire capture protocol takes approximately 24 hours, much of which is the overnight incubation for hybridization of capture probes. The protocol requires approximately 1.5 hours of hands-on time to process 12 samples in parallel, most of which is the series of washes performed after capturing the biotinylated probes on the avidin resin in the spin columns. We have greatly simplified the protocol from our previous work, which was performed in a custom-built microfluidic apparatus capable of processing one sample at a time, to the version described here, which uses disposable plastic spin columns in a small centrifuge with a 12-position rotor. This enables the protocol to be carried out in any well-equipped molecular biology laboratory, without any custom-built components. Temperature control is crucial; the most expensive single piece of equipment required is an incubator capable of maintaining at least 60°C, and large enough to house a small centrifuge and all of the necessary buffers and pipettes. We use an incubator with arm holes to allow a researcher to place his or her arms inside the incubator without opening a door, which helps maintain the temperature within the incubator. A photograph of the setup is illustrated in S9 Fig. Our previous method required approximately three days for the capture step alone, whereas the current approach based on spin columns allows preparation of the sequencing-ready library in two days. Further automation of the protocol is under development.

A notable advantage of our protocol for bacterial transcriptomics is the use of biotinylated capture probes that are synthesized directly from the genomic DNA of the bacterium of interest. Most other targeted capture techniques reported in literature rely upon capture probes or “baits” that comprise large libraries (ranging from hundreds to tens of thousands) of discrete synthetic DNA or RNA oligonucleotides, designed specifically to target sequences of interest. This approach is attractive, for example, for targeted capture of human exome sequences, where the same library of capture probes can be applied to many different studies. Synthetic capture probes may be an attractive option for organisms with very small genomes (*e*.*g*. RNA viruses), where the entire genome can be covered by a relatively small number of probes. Bacteria such as *K*. *pneumoniae*, however, have genomes that are both too large, and too highly variable (or mosaic) to be easily covered by a single set of synthetic capture probes: Kpn2146, has a genome of approximately 6 Mbp (including over 300 kbp on four plasmids); this would require a collection of 60,000 oligos 100 bases in length to completely cover the genome at 1X depth, and several times this number would be required to create a tiled capture array for more efficient capture. Given the large genomic variability between strains, and particularly since interesting genes are often located within mobile elements such as plasmids and genomic islands, a probe library synthesized for one strain may not be optimal for another. A study, for example, to compare the global gene expression of two or more strains becomes impractical if relying upon synthetic capture probe libraries. Our enzymatic synthesis technique, by contrast, allows creation of a random library covering the entire genome, in less than a day, for less than $20 per sample, from genomic DNA that can be produced quickly in large quantities from easily cultured organisms such as *K*. *pneumoniae*. Gene-targeted approaches have missed subsequently-discovered genes [49]; the unbiased genomic coverage achieved here can address unusual RNA genes or small protein genes that may only become delineated subsequent to the experiment. This method provides great savings over a library of chemically synthesized probes which requires careful design and several weeks to a few months of turnaround time, at a much higher cost per sample. Chemical synthesis of a custom probe library would only become cost-effective for large-scale studies (96 samples or more) with only a single, previously-sequenced bacterial strain of interest, where the same library can be used for many samples.

We note that our protocol bears some resemblance to the selective capture of transcribed sequences (SCOTS) technique that has been applied to microarray studies bacterial transcriptomics in infection models [50, 51]. The SCOTS technique relies upon a similar approach to preparing capture probes by biotinylating genomic DNA from the organism of interest. However, to achieve sufficient enrichment for microarray analysis, typically three rounds of hybridization and capture were performed, with an intermediate PCR amplification using a conserved primer. Besides being more time consuming, the intermediate PCR steps (up to 90 cycles across several rounds) may lead to additional bias. By contrast, we demonstrate here that a single round of capture-based enrichment is sufficient to dramatically improve coverage of pathogen sequences using RNA-seq.

## Conclusion

We have developed an improved method for targeted capture of bacterial transcripts from a mixed sample, in this case an infection model characterized by an overwhelming abundance of host RNA. The new protocol can be carried out in most well-equipped laboratories, and significantly improves the representation of bacterial genomes in an infection model. We established the capabilities of the technique using a multi-drug resistant *K*. *pneumoniae* isolate in a murine macrophage cell line. We demonstrated >100-fold enrichment of bacterial transcripts, allowing analysis of many genes that could not observed without enrichment, without dramatically biasing the relative expression between the captured and uncaptured libraries. Although this study was designed primarily to characterize the method, the results indicate expression of numerous drug resistance genes during the infection, with several drug-modifying enzymes expressed at high levels relative to the median of expressed genes, even in the absence of drugs. The results also suggest a role for the siderophore enterobactin early in the infection. Follow-up studies will further explore the biological significance of the expressed genes, including the effect of antimicrobial drugs on gene expression, as well as studying the coupled dynamics of host and pathogen gene expression in a more realistic infection model.

## Supporting Information

S1 Fig Capture increases representation of low-expressed genes. Box scatter plot of FPKM values for captured and uncaptured libraries, for timecourse infections with MOI of 10. The Capture samples reveal many more transcripts with low-level expression, that are not visible in the Uncaptured libraries.
(PNG)

S2 Fig Capture enrichment does not introduce bias in pure culture samples. (A) log_2_ gene FPKM counts were used to make scatter plots for the combined replicates of samples of culture treated with and without capture treatment. The R^2^ value of 0.94 was was calculated for the linear model fit of the scatter plot between the mentioned datasets, indicating high correlation between the captured and uncaptured datasets, with a high degree of linearity for highly expressed genes (indicating a lack of probe saturation effects). B-C) similarly the datasets used in A are used to compare with a dataset for infection sample which had a different gene expression profile. The R^2^ value computed for B and C are much lower than the culture captured and uncaptured sample in figure A.
(TIF)

S3 Fig Comparison of biological replicate experiments. This figure presents an array of scatter plots allowing comparison of individual pure culture experiments, including replicates of individual conditions, as well as comparisons between captured and uncaptured. For comparison, a set of infection samples (24 hour time point, with capture) are also included, showing that the infection experiments are dissimilar from the pure culture experiments, but more similar to each other. The plots along the main diagonal are histograms of FPKM for each condition.
(PDF)

S4 Fig Reproducibility of pure culture capture experiments. Each replicate of the samples tested were analyzed using the YAnTra pipeline to measure the FPKM counts of the gene feature. Sample to sample pairwise Pearson correlation coefficient was calculated for the overall gene expression profile of the samples. The distance matrix generated as 1-p is clustered and represented as a heatmap, with black indicating zero distance between samples, and bright yellow indicating maximum distance. The replicate samples analyzed form two prominent clusters: The samples sequenced coming from the culture samples for both captured and uncaptured, and the samples from the captured infection samples (24 hour time point) which were used to root the dendogram for clustering analysis.
(PNG)

S5 Fig Reproducibilty of capture enrichment experiments. As in S4 Fig, this figure presents a heatmap of sample to sample Pearson correlation coefficient of selected data sets after the hierarchical clustering, this time including the infection experiments (all time points, all replicates, with and without capture). The pure culture experiments (without capture) are included as an outgroup.
(PNG)

S6 Fig Capture improves coverage at early time points in infection. Mapping of reads to the plasmid pNDM-US, with and without capture, at early (2 hour) and late (24 hour) time points in infection.
(PNG)

S7 Fig Expression within genomic islands. Heatmap representation of gene expression (log2(FPKMs)) for genes located within genomic islands of Kpn2146.
(PNG)

S8 Fig Expression within genomic islands compared to median gene expression. Boxplots for gene expression distribution at 24 hr infection time point for the genomic islands. The black line shows the median gene expression of genes in non-island genes.
(PNG)

S9 Fig Experimental apparatus. Photographs of the equipment used for the spin column-based capture protocol carried out in an incubator with arm holes. The inset at the lower right shows a larger view of the spin column containing the monomeric avidin resin.
(JPG)

S1 Table Gene names for Fig 3B. Due to size constraints the names of individual genes are omitted from the rows of Fig 3B. The table provides a list of the locus tags with corresponding names from the annotation file, in the order in which they appear in the heat map.
(CSV)

S2 Table Read counts for infection experiments. The read counts for each sequencing experiment are provided, with the gene locus (corresponding to the annotation file in S4 Table) followed by the number of reads. The table includes three biological replicates for each experiment (time course and MOI experiments), with both captured and uncaptured data sets.
(CSV)

S3 Table FPKM for infection experiments. The cFPKM for each sequencing experiment are provided, with the gene locus (corresponding to the annotation file in S4 Table) followed by the calculated FPKM. The table includes three biological replicates for each experiment (time course and MOI experiments), with both captured and uncaptured data sets.
(CSV)

S4 Table Annotation file for Kpn2146. The table provides data on location, sense, and annotation for **each** gene referred to in S1, S2, and S3 Tables.
(CSV)
