# Supplementary figures and images for: A Rapid Spin Column-Based Method to Enrich Pathogen Transcripts from Eukaryotic Host Cells Prior to Sequencing
Source: PLoS One. 2016 Dec 21;11(12):e0168788. doi: 10.1371/journal.pone.0168788 (PMC5176299; doi:10.1371/journal.pone.0168788)

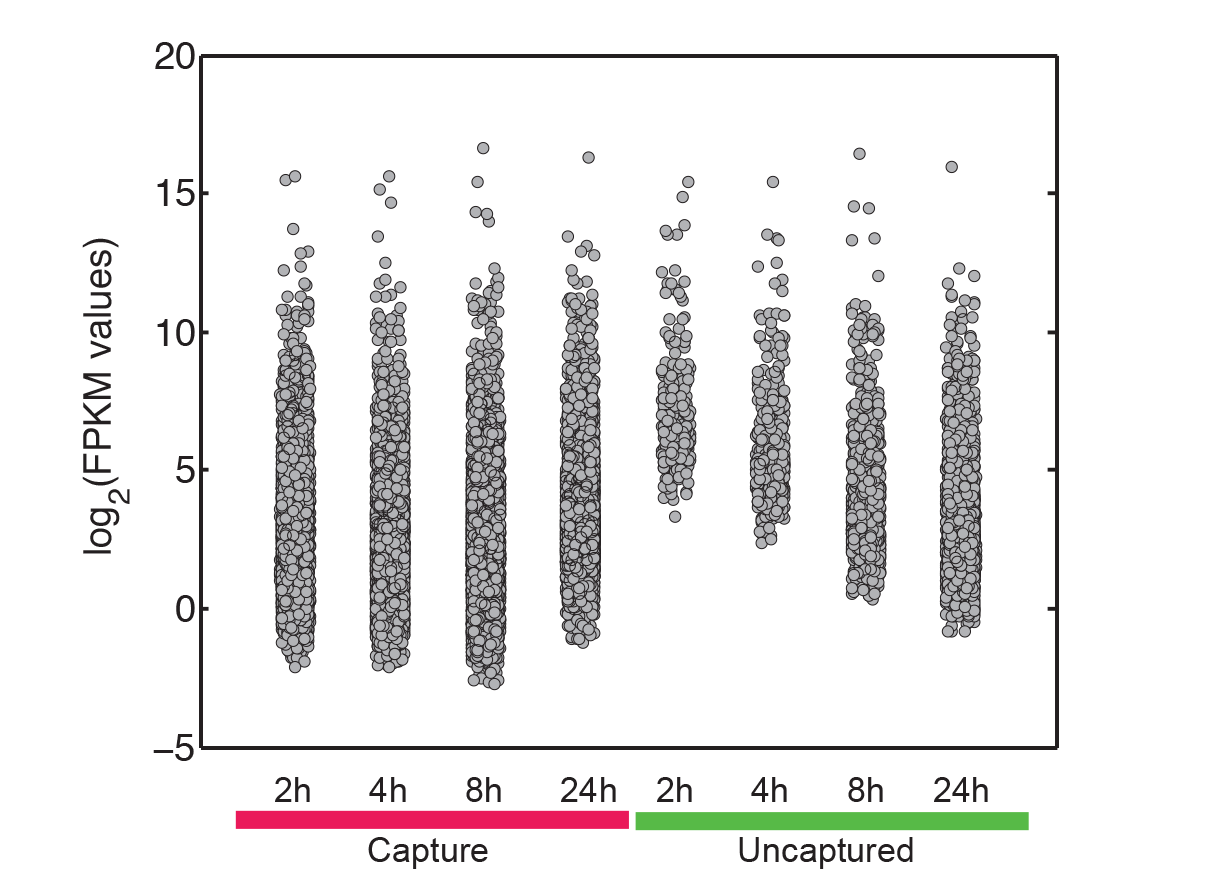

Supplement: S1 Fig — Box scatter plot of FPKM values for captured and uncaptured libraries, for timecourse infections with MOI of 10. The Capture samples reveal many more transcripts with low-level expression, that are not visible in the Uncaptured libraries. (PNG) [file pone.0168788.s001.png]

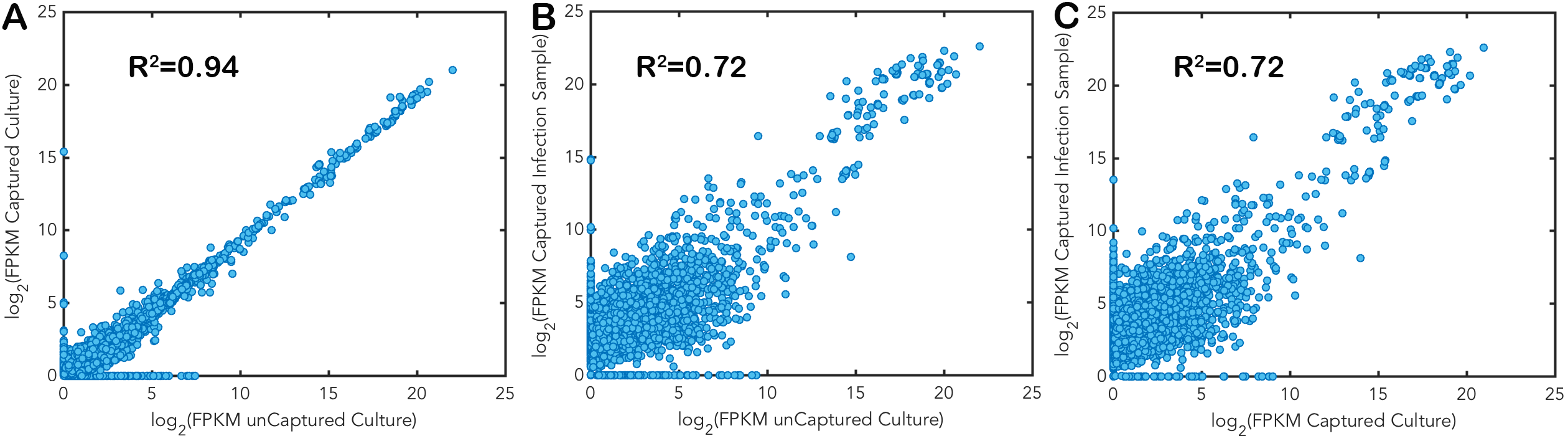

Supplement: S2 Fig — (A) log2 gene FPKM counts were used to make scatter plots for the combined replicates of samples of culture treated with and without capture treatment. The R2 value of 0.94 was was calculated for the linear model fit of the scatter plot between the mentioned datasets, indicating high correlation between the captured and uncaptured datasets, with a high degree of linearity for highly expressed genes (indicating a lack of probe saturation effects). B-C) similarly the datasets used in A are used to compare with a dataset for infection sample which had a different gene expression profile. The R2 value computed for B and C are much lower than the culture captured and uncaptured sample in figure A. (TIF) [file pone.0168788.s002.tif]

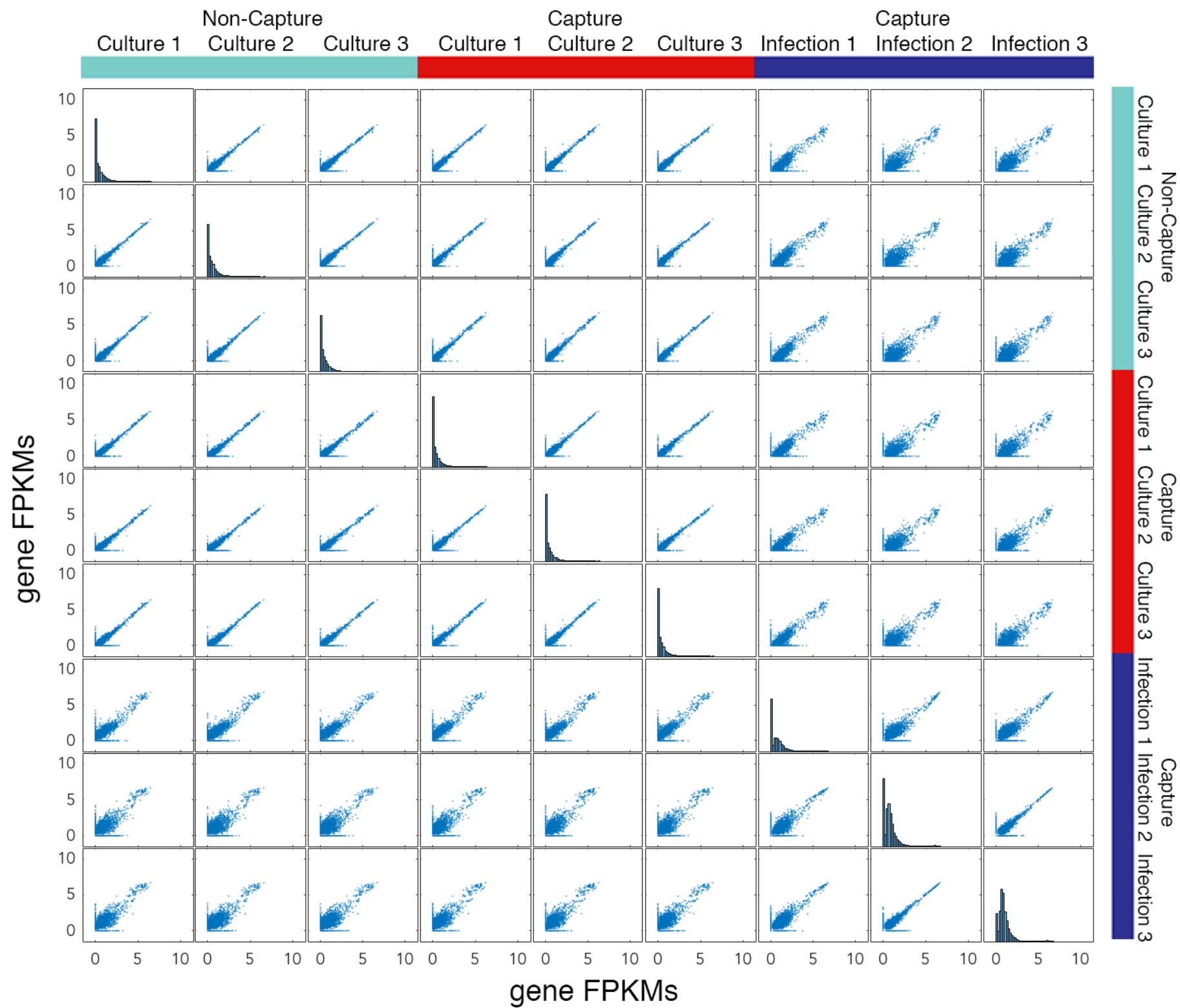

Supplement: S3 Fig — This figure presents an array of scatter plots allowing comparison of individual pure culture experiments, including replicates of individual conditions, as well as comparisons between captured and uncaptured. For comparison, a set of infection samples (24 hour time point, with capture) are also included, showing that the infection experiments are dissimilar from the pure culture experiments, but more similar to each other. The plots along the main diagonal are histograms of FPKM for each condition. (PDF) [file pone.0168788.s003.pdf]

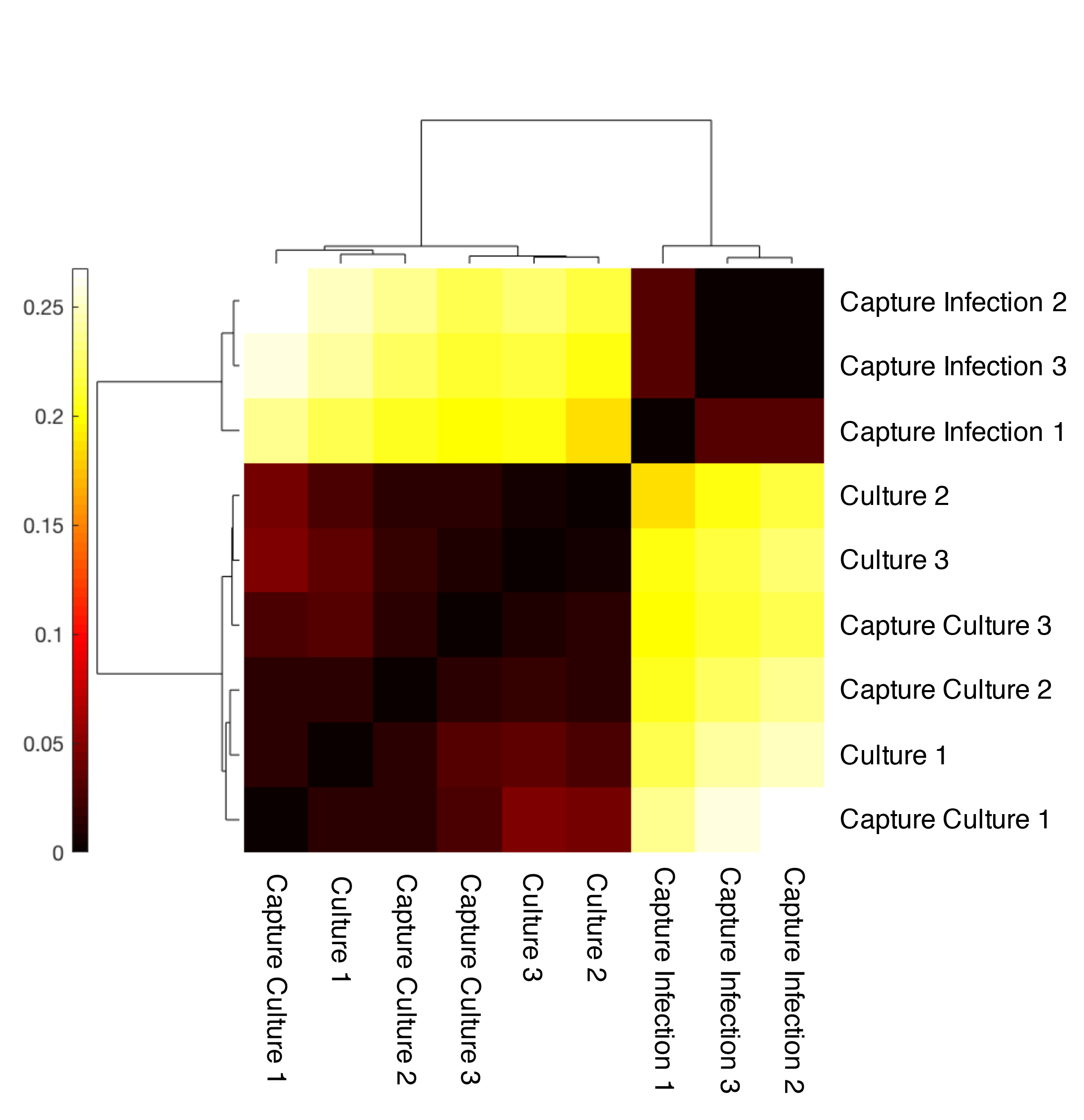

Supplement: S4 Fig — Each replicate of the samples tested were analyzed using the YAnTra pipeline to measure the FPKM counts of the gene feature. Sample to sample pairwise Pearson correlation coefficient was calculated for the overall gene expression profile of the samples. The distance matrix generated as 1-p is clustered and represented as a heatmap, with black indicating zero distance between samples, and bright yellow indicating maximum distance. The replicate samples analyzed form two prominent clusters: The samples sequenced coming from the culture samples for both captured and uncaptured, and the samples from the captured infection samples (24 hour time point) which were used to root the dendogram for clustering analysis. (PNG) [file pone.0168788.s004.png]

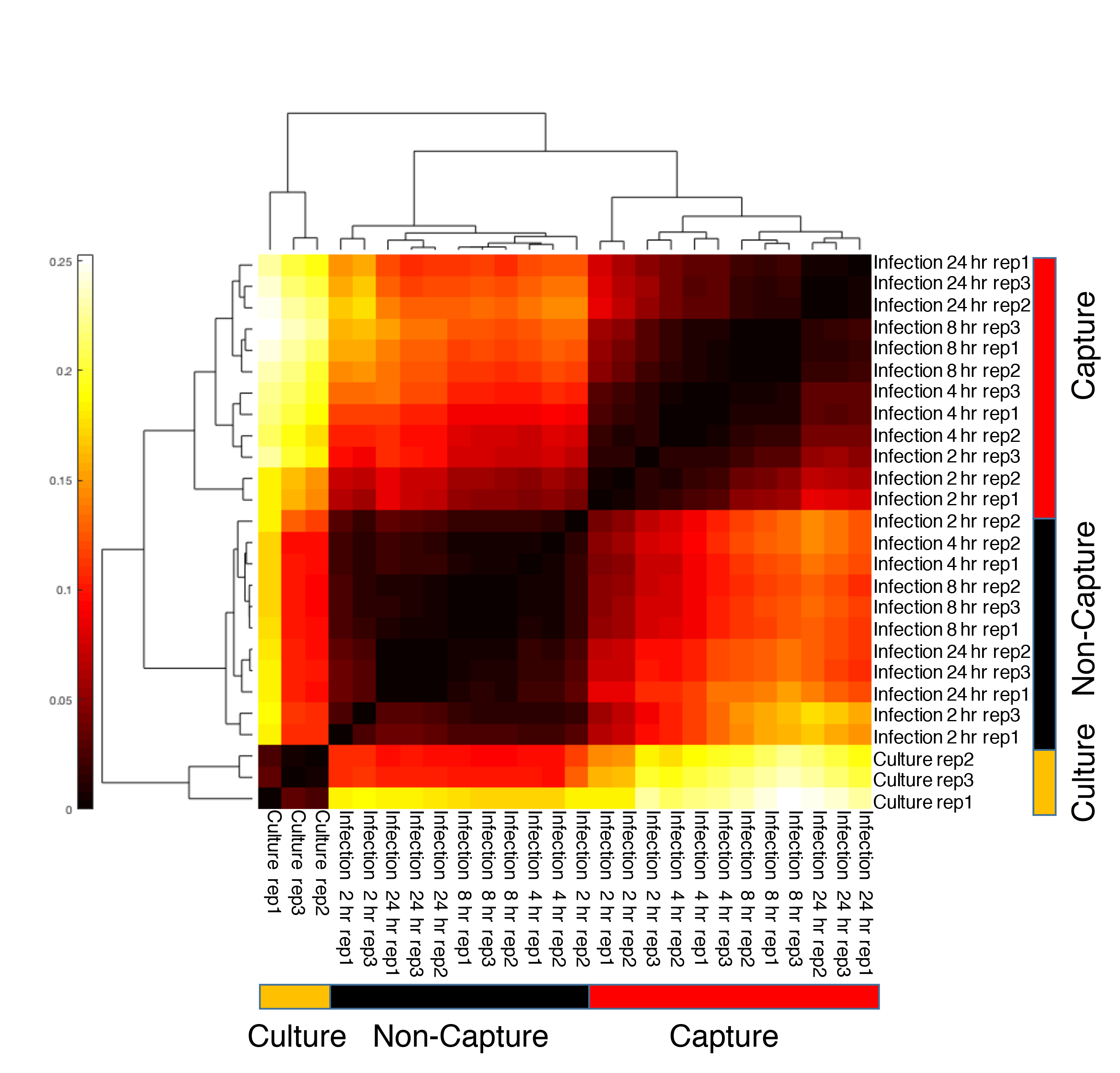

Supplement: S5 Fig — As in S4 Fig, this figure presents a heatmap of sample to sample Pearson correlation coefficient of selected data sets after the hierarchical clustering, this time including the infection experiments (all time points, all replicates, with and without capture). The pure culture experiments (without capture) are included as an outgroup. (PNG) [file pone.0168788.s005.png]

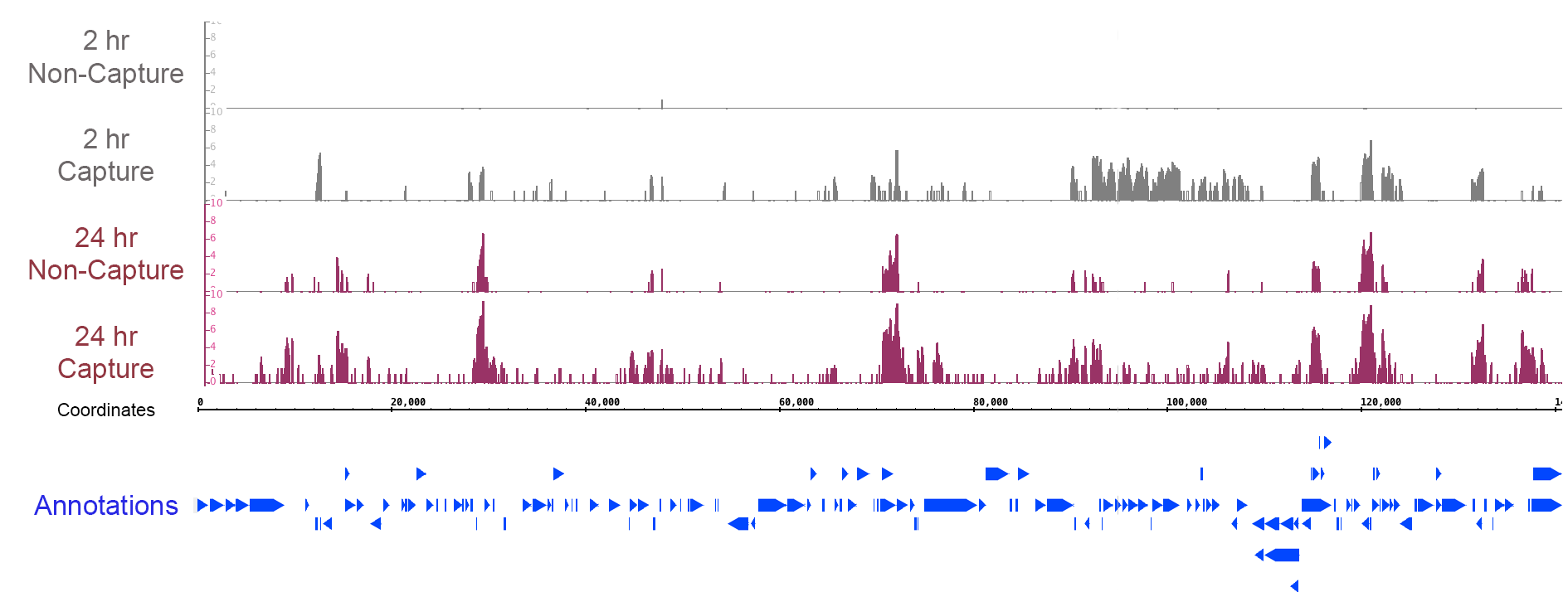

Supplement: S6 Fig — Mapping of reads to the plasmid pNDM-US, with and without capture, at early (2 hour) and late (24 hour) time points in infection. (PNG) [file pone.0168788.s006.png]

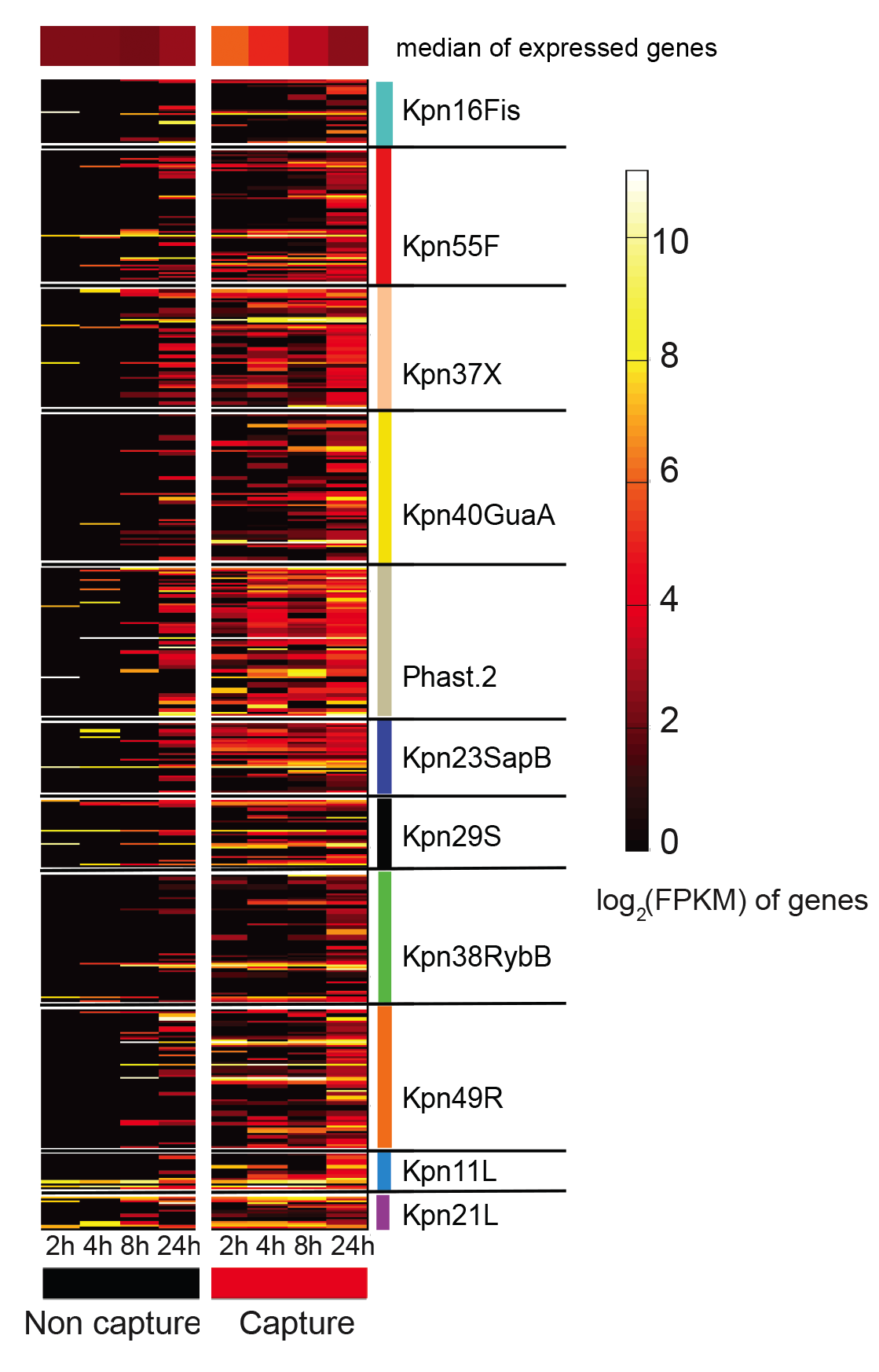

Supplement: S7 Fig — Heatmap representation of gene expression (log2(FPKMs)) for genes located within genomic islands of Kpn2146. (PNG) [file pone.0168788.s007.png]

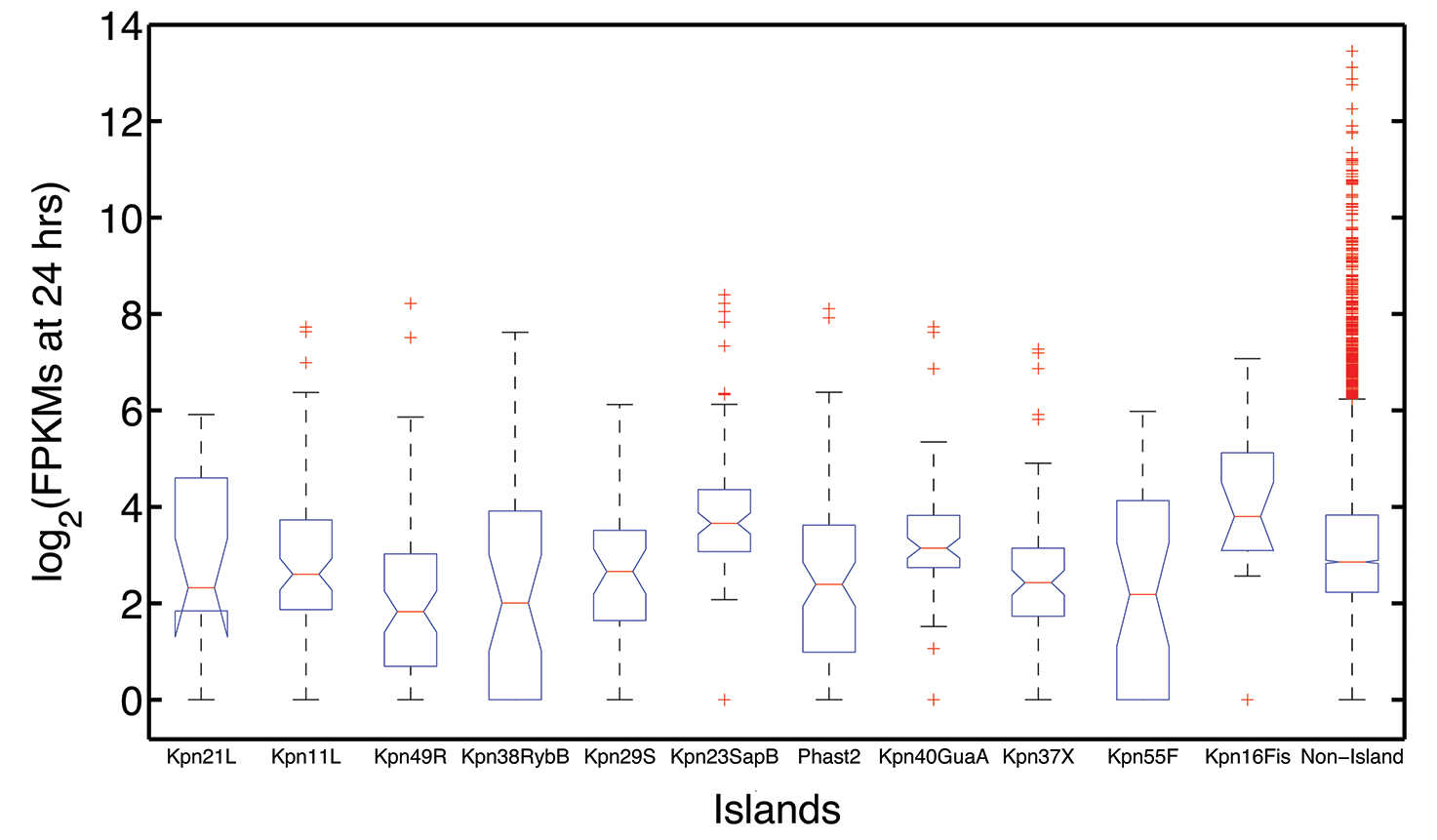

Supplement: S8 Fig — Boxplots for gene expression distribution at 24 hr infection time point for the genomic islands. The black line shows the median gene expression of genes in non-island genes. (PNG) [file pone.0168788.s008.png]

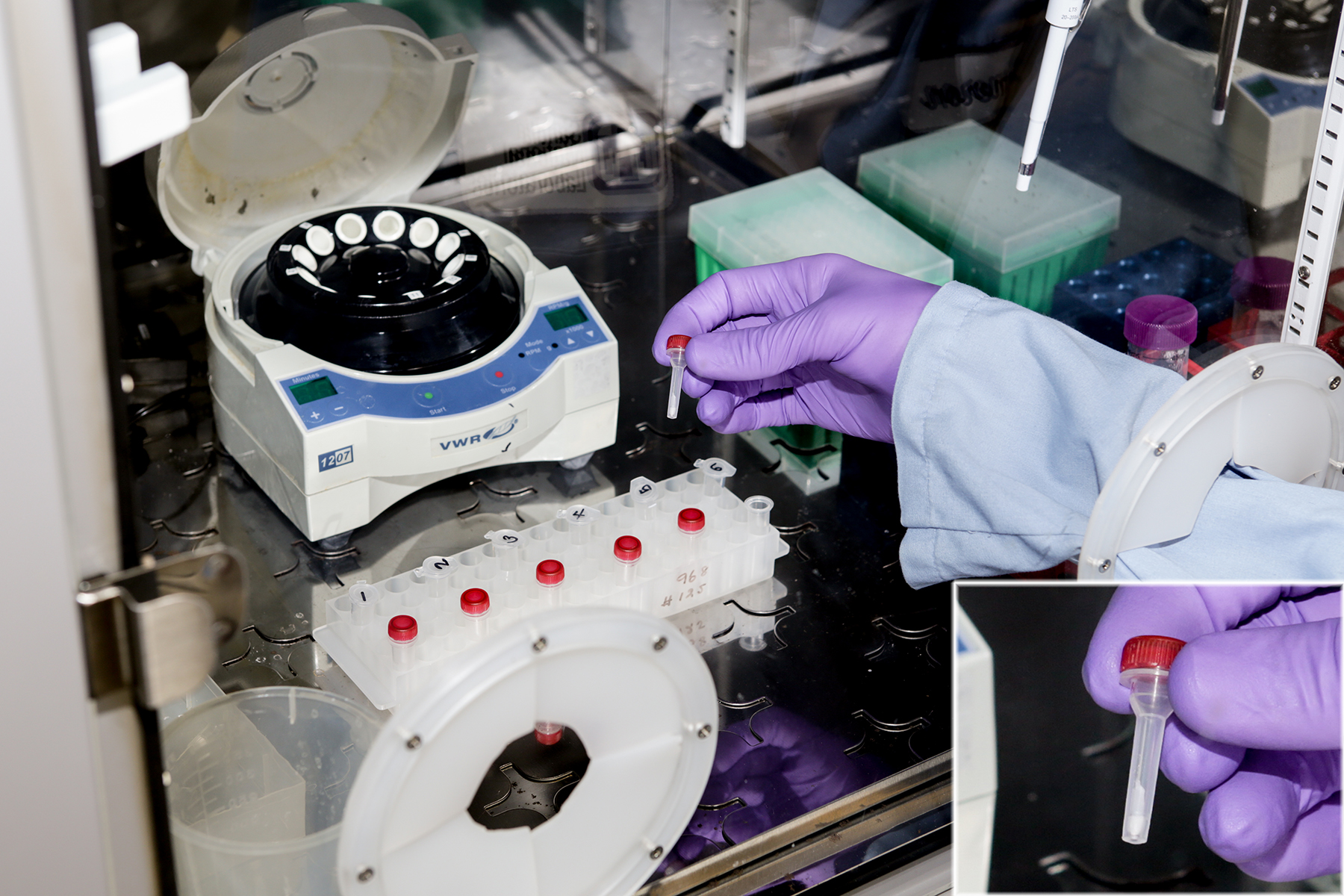

Supplement: S9 Fig — Photographs of the equipment used for the spin column-based capture protocol carried out in an incubator with arm holes. The inset at the lower right shows a larger view of the spin column containing the monomeric avidin resin. (JPG) [file pone.0168788.s009.jpg]
